# Alternative Sigma Factor B in Bovine Mastitis-Causing *Staphylococcus aureus*: Characterization of Its Role in Biofilm Formation, Resistance to Hydrogen Peroxide Stress, Regulon Members

**DOI:** 10.3389/fmicb.2019.02493

**Published:** 2019-11-07

**Authors:** Sirirak Supa-amornkul, Paninee Mongkolsuk, Pijug Summpunn, Pongkorn Chaiyakunvat, Warisara Navaratdusit, Chutima Jiarpinitnun, Soraya Chaturongakul

**Affiliations:** ^1^Mahidol International Dental School, Faculty of Dentistry, Mahidol University, Bangkok, Thailand; ^2^Department of Microbiology, Faculty of Science, Mahidol University, Bangkok, Thailand; ^3^Food Technology and Innovation Research Center of Excellence, School of Agricultural Technology, Walailak University, Nakhon Si Thammarat, Thailand; ^4^Department of Chemistry and Center for Innovation in Chemistry (PERCH-CIC), Faculty of Science, Mahidol University, Bangkok, Thailand

**Keywords:** *Staphylococcus aureus*, alternative sigma factor B, staphylococcal bovine mastitis, transcriptome, bacterial stress response

## Abstract

This study examines treatments of the bacterial pathogen *Staphylococcus aureus*, namely, in the context of its being a major cause of subclinical bovine mastitis. Such infections caused by *S. aureus* among dairy cows are difficult to detect and can easily become chronic, leading to reduced productivity and large losses for dairy manufacturers. In this study, the role of alternative sigma factor B (σ^B^), which has been shown to be a global regulator for *S. aureus* infections, was explored in a mastitis-causing *S. aureus* strain, RF122. For comparison with the wild-type strain, a *sigB* null (Δ*sigB*) mutant was constructed and analyzed for its phenotypes and transcriptome. Our study found that σ^B^ is essential for biofilm formation as the Δ*sigB* mutant strain produced significantly less biofilm than did the wild-type strain at 48 h. σ^B^ is involved in response to H_2_O_2_ stress. However, σ^B^ plays a minor or no role in resistance to antiseptics (e.g., povidone-iodine and chlorhexidine), resistance to tested antibiotics, hemolysin activity, and invasion ability. RNA sequencing identified 225 σ^B^-dependent genes, of which 171 are positively regulated and 54 are negatively regulated. The identified genes are involved in stress response, pathogenesis, and metabolic mechanisms. Quantitative TaqMan RT-PCR was performed to verify the RNA sequencing results; i.e., σ^B^ is a positive regulator for *asp23, sarA, katA, yabJ, sodA, SAB2006c*, and *nrdD* expressions. In the RF122 strain, σ^B^ plays a role in biofilm formation, general stress response (e.g., H_2_O_2_), and regulation of virulence factors and virulence-associated genes.

## Introduction

Bovine mastitis is an inflammation of the mammary gland (Viguier et al., 2009), which negatively impacts the dairy industry, leading to economic losses due to reduced milk production and increased treatment expenses (Wall et al., 2005; Le Maréchal et al., 2011b). *Staphylococcus aureus* is one of the most prevalent causative agents of subclinical and clinical mastitis (Dego et al., 2002; Azizoglu et al., 2013). However, unlike clinical mastitis, subclinical mastitis shows few visible symptoms in infected cows (Viguier et al., 2009; Le Maréchal et al., 2011a). The inability to rapidly detect subclinical mastitis leads to a high prevalence of such infections being observed in dairy farms (Gruet et al., 2001). Subclinical mastitis is caused by several species of bacteria including *S. aureus* (Gruet et al., 2001). Treatment of *S. aureus* subclinical mastitis can be more difficult because *S. aureus* is known to invade phagocytes where the concentration of antibiotics is sublethal. Persistent *S. aureus* can lead to deep-seated abscesses, which further serve as a niche for chronic infection (Hébert et al., 2000; Gruet et al., 2001; Brouillette and Malouin, 2005; Pieterse and Todorov, 2010). Antibiotic-resistant *S. aureus*, particularly relating to β-lactam drugs, has been reported among mastitis-causing strains (Gruet et al., 2001). Thus, alternative treatments (e.g., anti-CD40 monoclonal antibody) or novel drug targets (e.g., transcription factors) are needed strategies to combat bovine mastitis (Wallemacq et al., 2012).

In bacteria, alternative sigma (σ) factors are transcription factors involved in promoter recognition and RNA polymerase recruitment (Schulthess et al., 2011). They play crucial roles in regulation of gene expression in response to change in environments (Schulthess et al., 2011). The most studied alternative σ factor in Firmicutes (e.g., *Bacillus* and *Staphylococcus* spp.) is σ^B^. σ^B^-regulated genes include those involved in general stress response, virulence, capsule formation, and biofilm formation (Nicholas et al., 1999; Hecker et al., 2007; Meier et al., 2007; Kim et al., 2008; Cebrián et al., 2009; Lauderdale et al., 2009; Schulthess et al., 2009, 2011). In *S. aureus*, σ^B^ controls expression of virulence genes via regulation of accessory regulator A (*sarA*) and accessory gene regulator (*agr*), which regulate production of extracellular proteins (secreted enzymes and toxins) and cell surface proteins (Bischoff et al., 2001). This study aimed to further explore the roles of σ^B^ and the identification of σ^B^ regulon in mastitis-causing *S. aureus* strains. The results validated the potential of σ^B^ as an alternative therapeutic target for *S. aureus*-induced subclinical bovine mastitis.

## Methods

### Bacterial Strains

Mastitis-causing *S. aureus* strain RF122 (received as a gift from Professor Vivek Kapur, Penn State University) was used as a wild-type strain. *Escherichia coli* strain DH5α was used to prepare competent cells for plasmid propagation in the plasmid construction step. *S. aureus* was cultured in brain heart infusion (BHI) or tryptic soy medium (Difco), and *E. coli* was cultured in Luria–Bertani (LB) medium (Difco) at 37°C with 200 rpm agitation. For long-term preservation, 20% sterilized glycerol was added into overnight culture and stored at −80°C.

### Mutant Construction

A pKSV7 Δ*sigB* plasmid was constructed using the splicing by overlapping extension polymerase chain reaction (SOE-PCR) method (Wiedmann et al., 1998; Yakhnin and Babitzke, 2004). The SOE-PCR primers for mutant construction are listed in Supplementary Table 1. A pKSV7 Δ*sigB* plasmid (717 bp in-frame deletion) was transformed into *E. coli* DH5α for propagation. Competent cells of *S. aureus* and electroporation were performed as described in Monk et al. (2012). The allelic exchange mutagenesis was carried out following the previously reported procedures of Yakhnin and Babitzke (2004). Deletion of *sigB* in Δ*sigB* mutant was confirmed by DNA sequencing (Macrogen, Korea). Growth of wild type and Δ*sigB* in tryptic soy broth (TSB) media at 37°C with 200 rpm agitation was determined every 2 h for 12 h.The growth experiments were performed in triplicates.

### RNA Sequencing and Data Analysis

The post-exponential phase samples, defined as an OD_600_ of 1.0 with an additional 3 h incubation of *S. aureus* wild-type and Δ*sigB* mutant strains, were collected for RNA sequencing (RNA-Seq). RNAprotect (Qiagen) was added to bacterial cultures to stop cellular activity and to stabilize RNA. RNA was extracted using TRIzol (Invitrogen) followed by an RNeasy Mini Kit (Qiagen). Total RNA samples were sent to Molecular Genomics (Singapore) for RNA-Seq. RNA quality and quantity were determined using Agilent 2100 Bioanalyzer (Agilent Technologies, Santa Clara, CA) and Agilent RNA 6000 Pico Kit (Agilent Technologies, Santa Clara, CA). A HiSeq 2500 sequencer (Illumina) was selected as a platform for RNA-Seq in this study. The quality of output sequences was determined by FastQC program version 0.11.7 (https://www.bioinformatics.babraham.ac.uk/projects/fastqc/). Sequences with a Phred quality score of more than 30 were used for further analysis. Genome sequence and gene functions of *S. aureus* RF122 from NCBI (ASM900v1) were used as reference information to a generated bowtie index using Bowtie2 (Langmead et al., 2018). TopHat 2.1.1 was employed for alignment of RNA sequences in reference to the bowtie index (Kim et al., 2013). HTSeq-count was used to count reads for differential expression analysis (Pyl et al., 2014). DeSeq2 package version 3.2 in R program was also used to test differential expression based on negative binomial generalized linear models (Love et al., 2014). The cut-off values of expression fold change (wild type vs. Δ*sigB*) determined by DeSeq2 were set at 2, i.e., ≥2 for upregulated genes and −2 or less for downregulated genes, and *p*-adjusted (*p*adj) values <0.05 were reported as differentially expressed in wild type and in the Δ*sigB* mutant strains.

### qRT-PCR Analysis

Total RNA was extracted from the post–exponential phase culture using TRIzol reagent (Invitrogen), Mini-BeadBeater (Bio-Spec Products), and RNeasy Mini Kit (Qiagen). RNA yield was determined by NanoDrop (ND-1000, NanoDrop Technologies, Inc.). Quantitative reverse transcription-PCR (qRT-PCR) was performed on seven selected genes [*asp23, sarA*, catalase gene (*katA*), *yabJ*, aldehyde dehydrogenase gene (*SAB2006c*), *sodA*, and *nrdD*] to verify σ^B^-dependence identified by RNA-Seq using LightCycler 480 (Roche). Expression levels were normalized with those of the housekeeping gene *rpoB*. For each RNA sample, qRT-PCR was performed in duplicate reactions. Experiments were performed in biological triplicates.

### Phenotypic Characterization of Δ*sigB* Mutant Strain

Antibiotic and Antiseptic Susceptibilities in *S. aureus* Wild Type and Δ*sigB* Strains

*S. aureus* wild type and Δ*sigB* mutant strains were grown in TSB at 37°C overnight. Each culture was diluted in 0.85% sterile saline to a density equivalent to 0.5 McFarland standards (approximately OD_625_ of 0.08–0.13) [Clinical and Laboratory Standards Institute (CLSI, 2012)] and spread onto Mueller Hinton (MH) agar plates using a sterile cotton swab. The antibiotic disks were aseptically placed on the surface of agar. The plates were incubated at 37°C for 18 h, and the inhibitory zones were measured by a digital Vernier caliper. The results were interpreted according to the Clinical and Laboratory Standards Institute CLSI (2012). The antibiotic disks (Oxoid, UK) used were ampicillin (10 μg), penicillin (10 μg), oxacillin (1 μg), cefoxitin (30 μg), ceftiofur (30 μg), cephalothin (30 μg), tetracycline (30 μg), streptomycin (10 μg), erythromycin (15 μg), sulfamethoxazole with trimethoprim (25 μg), sulfamethoxazole (25 μg), and novobiocin (30 μg). The interpretative zone diameters of ceftiofur and streptomycin are not stated in CLSI criteria. Based on previously reported results, susceptibility of ceftiofur was considered ≥21 mm (Soares et al., 2014) and ≤16 mm for resistance to streptomycin (Shittu and Lin, 2006). These antibiotics were selected in this study due to their frequent use in bovine mastitis treatment (Azizoglu et al., 2013).

Iodine and chlorhexidine are typical antiseptics used in dairy farms; their minimum inhibitory concentrations (MICs) and minimum bactericidal concentrations (MBCs) were determined based on the protocol described as follows. Five hundred microliters of OD_600_ 0.4 culture was added into 50 ml of TSB, and the culture was incubated for 6 h. The culture was diluted into MH broth to achieve 5 × 10^5^ colony-forming units (CFUs)/ml and used for the experiments. Ten percent of povidone-iodine solution containing 1% of available iodine (Azizoglu et al., 2013) was used to prepare the concentrations of iodine. The final concentrations of iodine were 0.0001, 0.0005, 0.001, 0.005, 0.01, 0.05, 0.1, 0.15, 0.2, and 0.25%. The treated bacterial culture was grown at 37°C for 18 h. Growth was measured by monitoring OD_600_, and MIC was reported as the lowest antiseptic concentration which inhibits *S. aureus* growth. Concentrations that inhibited growth were streaked out onto tryptic soy agar (TSA) plates to determine the lowest antiseptic concentration which completely killed *S. aureus* or MBC.

The same protocol was used to determine the MIC and MBC for chlorhexidine. The final concentrations of chlorhexidine diacetate were 0.00001, 0.00002, 0.00005, 0.0001, 0.0002, and 0.0004%.

### Oxidative Stress Survival Assay

Both wild type and Δ*sigB* mutant strains were grown to the post-exponential phase. Culture CFUs were determined and recorded by serial dilution and spreading onto TSA. One milliliter of culture was mixed with cumene hydroperoxide (CHP; Sigma) at a final concentration of 7.5 mM. The untreated and treated bacterial culture mixtures were incubated at 37°C, with 200 rpm agitation, for 15, 30, and 60 min. At each time point, samples were spread onto TSA to determine CFU. Survival abilities of wild-type and Δ*sigB* mutant strains were compared from “log reduction,” which was calculated from log CFU per milliliter of CHP-untreated minus log CFU per milliliter of CHP-treated for each time point. For H_2_O_2_ survival, 1 ml of post-exponential phase culture was mixed with H_2_O_2_ at 880 mM final concentration and incubated at 37°C, with 200 rpm agitation, for 10 and 20 min. CFU was determined, and survival abilities were compared from log reductions between untreated and treated samples at each time point. All oxidative stress survival assays were repeated at least three times.

### Biofilm Formation

To quantify the biofilm formation of *S. aureus*, a microplate assay was performed based on the previously reported protocol (Mitchell et al., 2010) with slight modifications as follows. A colony of wild-type or Δ*sigB* mutant strain was inoculated into TSB containing 0.5% glucose and incubated overnight at 37°C, 200 rpm. The culture was diluted to 0.5 McFarland in TSB containing 0.5% glucose and transferred into a 96-well flat-bottom microtiter plate containing a fresh TSB medium in the ratio 1:1. The plates were incubated at 37°C for 24 and 48 h. Culture broth was discarded, and the wells (with attached biofilm) were washed three times with 200 μl of phosphate buffer saline (PBS) pH 7.4. The wells were stained for 30 min with 1% of crystal violet, washed with 100 μl of water to remove excess staining solution, and let dry. Two hundred microliters of 30% acetic acid solution was added to dissolve the crystal violet. The resulting solution was measured at OD_560_ using the microplate reader (Wallac Victor 1420, PerkinElmer, USA). Biofilm formation was evaluated in three replicates.

### Hemolysis Assay

Sheep blood was washed three times in cold PBS and centrifuged at 4,000 rpm for 10 min at 4°C (Rotina 380R, Hettich, UK) (Lauderdale et al., 2009). Sheep red blood cells were then diluted to a final concentration of 1%. The post-exponential phase cultures were 2-fold serially diluted for seven times starting from 10^9^ to 5 × 10^5^ with PBS pH 7.4. One hundred microliters of undiluted and diluted culture were mixed with 900 μl of 1% sheep red blood cells and incubated at 37°C for 30 min. The mixtures were centrifuged at 4,000 rpm for 10 min at 4°C (Centrifuge 5417R, Eppendorf, USA). Hemolytic activity was measured at OD_545_. Positive control was defined as a complete lysis of red blood cells by 1% Triton-100. The hemolytic unit was defined as the inverse of dilution when the OD_545_ reading was more than 50% of complete hemolysis [(positive control reading – negative control reading)/2] (Menzies and Kernodle, 1994).

### Cell Invasion Assay

A549 (human lung epithelial cell line) was used to study the invasion assay. In routine A549 maintenance, Dulbecco's Modified Eagle Medium (DMEM) (Gibco) with 10% fetal bovine serum (FBS), 1% final concentration of penicillin/streptomycin, and 5 mM of l-glutamine was used. The cell invasion assay was performed as previously described (Liang et al., 2006). In brief, ~2 × 10^5^ cells of A549 (human lung epithelial cell line) were seeded in 24-well plates and incubated for 18 h at 37°C, 5% CO_2_. A549 cells were infected by *S. aureus* wild-type or Δ*sigB* mutant strain by adding 0.5 ml DMEM containing 5 × 10^5^ CFU of bacteria. The multiplicity of infection value used in this study was 2.5 (2.5 bacterial cells: 1 A549 cell). The plate was incubated at 37°C for 10 min followed by centrifugation at 100 × *g* for 5 min and continued incubation for 2 h. The culture medium was removed and discarded. Subsequently, the A549-infected cells were washed three times with PBS, pH 7.4. To eliminate extracellular bacteria, 1 ml of DMEM supplemented with 10% FBS, 100 μg/ml gentamicin, and 5 μg/ml lysostaphin was added to each well. The plate was incubated for 1 h; then the supernatant was removed. All wells were washed three times with PBS and then trypsinized by 0.25% trypsin-EDTA. After 5 min, the cells in each well were carefully collected and kept on ice. Four hundred microliters of 0.025% Triton X-100 was added to each tube. The numbers of released bacteria from A549 were determined by CFU on TSA. The percentage of the invasion of Δ*sigB* mutant was calculated and compared with that of the wild-type strain, which was considered to be 100% (Liang et al., 2006).

### Statistical Analysis

An independent *t*-test and Mann–Whitney *U*-test were used to analyze data for phenotypic characterization and qRT-PCR. Statistical significance was reported when the *p* ≤ 0.05.

## Results

**Figure 1:**
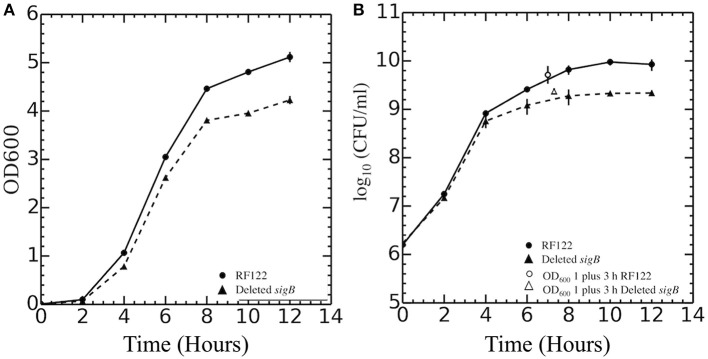
Growth curves of *Staphylococcus aureus* RF122 and Δ*sigB* mutant strains. **(A)** Relationship between OD_600_ of each strain and time of incubation. **(B)** Relationship between log_10_ colony-forming units (CFU) per milliliter of each strain and time of incubation. The experiment was performed in three biological replicates.

In this study, Δ*sigB* strain in RF122 was constructed, and the role of σ^B^ in stress response was assessed by comparing the phenotypes of the wild type to those of the mutant. In contrast to the previous studies, our study show that the growth of the Δ*sigB* mutant strain was different from the wild type (Figure 1). Although the specific growth rates of wild type (0.41 h^−1^) and Δ*sigB* mutant (0.44 h^−1^) were not different, the maximum numbers of CFUs reached in batch cultures were significantly different (*p* = 0.04). The average maximum CFU reached by wild type was 1.1 × 10^10^ CFU/ml while that of Δ*sigB* mutant was 2.5 × 10^9^ CFU/ml. In fact, CFU values during the post-exponential phase and early stationary phase (*t* = 6, 8, 10, 12) were significantly higher in the wild-type strain. Macroscopically, colonies of the Δ*sigB* mutant strain on TSA showed less production of the yellow pigment, potentially staphyloxanthin, when compared to the wild-type strain (Supplementary Figure 1). Wild-type and Δ*sigB* mutant strains were further characterized for their transcriptomes and phenotypes. The following section lists the key findings of this study.

### σ^B^ Regulon in RF122 Strain

**Figure 2:**
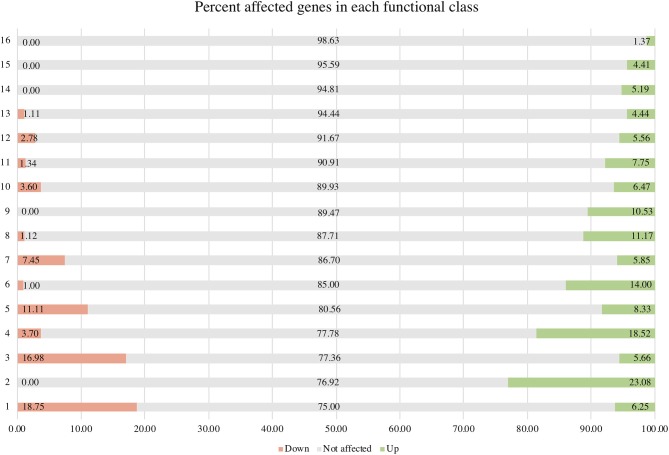
Percentage of positively and negatively σ^B^-regulated genes in each functional class. Functional classes: 1 = central intermediary metabolism; 2 = purines, pyrimidines, nucleoside, and nucleotides; 3 = amino acid biosynthesis; 4 = cellular processes; 5 = fatty acid and phospholipid metabolism; 6 = cellular proteins; 7 = transporter and binding proteins; 8 = cell envelope; 9 = transcription; 10 = energy metabolism; 11 = hypothetical protein; 12 = biosynthesis of cofactors, prosthetic groups, and carriers; 13 = regulatory functions; 14 = mobile and extrachromosomal element function; 15 = unknown function; and 16 = DNA metabolism.

σ^B^ actively functions throughout the growth phase and peaks at the early stationary phase (Senn et al., 2005; Pané-Farré et al., 2006; Schulthess et al., 2009). Using isogenic Δ*sigB* and RF122 wild-type strains grown to post–exponential phases, we performed RNA-Seq to determine the members of σ^B^ regulon. The raw sequence data (.fastq files) of all samples and summary of RNA-Seq results were submitted to the SRA database under SRA accession number PRJNA548307. The overview of the RNA-Seq result is shown in Supplementary Table 2. We identified 171 positively regulated genes with higher transcription levels in the wild-type strain than in the Δ*sigB* mutant strain (fold change ≥ 2) (Supplementary Table 3). Fifty-four genes were expressed in higher levels in the Δ*sigB* mutant strain than in the wild type (fold change ≤ −2) and were defined as negatively σ^B^-regulated genes (Supplementary Table 3). In *S. aureus, sigB* operon is composed of *rsbU, rsbV, rsbW*, and *sigB* (Hecker et al., 2007). The expression of genes in the *sigB* operon was lower in the Δ*sigB* mutant compared to the wild-type strain, but only the expression levels of the *sigB* and *rsbW* genes differed more than 2-fold with *p*adj being <0.05 (Supplementary Table 3). All affected genes were classified into 16 classes from 17 functional classes of genes in *S. aureus*. The only one functional class not affected by σ^B^ was signal transduction. The highest affected class was the central intermediary metabolism class (Table 1). The percentage of positively and negatively σ^B^-regulated genes in each functional class is shown in Figure 2. Most of the functional classes were affected positively or negatively by σ^B^ (Figure 2). Five functional classes in which all affected genes were positively σ^B^-regulated were DNA metabolism, unknown function, mobile and extrachromosomal element functions, transcription, and purines, pyrimidines, nucleosides, and nucleotides (Figure 2). Among the upregulated genes, we found genes involved in general stress response and virulence such as alkaline shock protein (*asp23*), accessory regulator A (*sarA*), capsule polysaccharide synthesis enzyme (*cap*), hemolysin, and translation initiation inhibitor (*yabJ*). Detoxifying enzymes are catalase and superoxide dismutase (*sodA*). Several genes are involved in carbohydrate metabolism (glycolysis/gluconeogenesis) as well as fatty acid metabolism such as trimethylamine dehydrogenase and aldehyde dehydrogenase. Genes involved in cell wall synthesis, *murE* and *murD*, were also positively regulated by σ^B^.

**Table 1:** Number for σ^B^-regulated genes in each functional class.

**No**.	**Functional class**	**Total gene in class**	**Number of affected genes**	**% affected**
1	DNA metabolism	73	1	1.37
2	Unknown function	68	3	4.41
3	Mobile and extrachromosomal element functions	135	7	5.19
4	Regulatory functions	90	5	5.56
5	Biosynthesis of cofactors, prosthetic groups, and carriers	36	3	8.33
6	Hypothetical proteins	748	68	8.96
7	Energy metabolism	139	14	10.07
8	Transcription	19	2	10.53
9	Cell envelope	358	44	12.29
10	Transport and binding proteins	188	25	13.30
11	Cellular	100	15	15.00
12	Amino acid biosynthesis	53	12	18.87
13	Fatty acid and phospholipid metabolism	36	7	19.44
14	Cellular processes	54	12	22.22
15	Purines, pyrimidines, nucleosides, and nucleotides	13	3	23.08
16	Central intermediary metabolism	16	4	25.00

**Figure 3:**
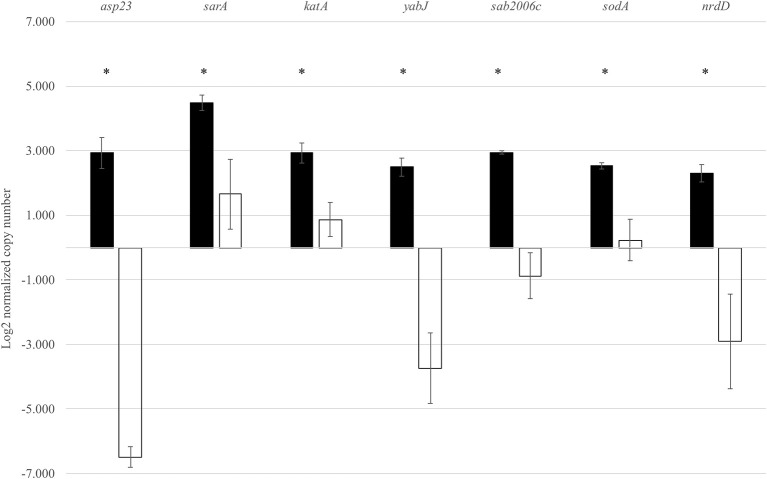
Expression of seven selected genes in both the wild type and Δ*sigB* mutant determined by qRT-PCR. Differences in mRNA levels of all genes are statistically significant when compared between RF122 wild-type (black) and Δ*sigB* mutant (white) strains. *significant difference between wild-type and Δ*sigB* mutant strains at *p* < 0.05.

In order to validate the RNA-Seq results, seven genes identified by RNA-Seq to be σ^B^ dependent were selected for qRT-PCR. These genes were *asp23, sarA, katA, yabJ, sodA, SAB2006c*, and *nrdD*. Expression levels of these genes were normalized to the expression level of a housekeeping gene *rpoB*. As expected, the wild-type strain showed significantly higher levels of *asp23, sarA, katA, yabJ, SAB2006c, sodA*, and *nrdD* expressions when compared to the Δ*sigB* mutant (*p* < 0.05). The results correlated to the data obtained from RNA-Seq (Figure 3). The fold change from qRT-PCR of each gene was determined and compared with the RNA-Seq result (Supplementary Table 3). All selected genes showed fold change values of more than 1.5-fold, the cutoff value to determine the differential expression in qRT-PCR. Based on the results from qRT-PCR, the *asp23* gene exhibited the highest fold change of 7.14 while the catalase gene showed the lowest fold change of 1.53 (Supplementary Table 4). However, the highest fold change from these seven genes from RNA-Seq was *sodA* (13.80), contradicting with the fold change result from qRT-PCR (1.60) (Supplementary Table 4).

### σ^B^ in Mastitis-Causing *S. aureus* May Be Involved in Susceptibility to Cell-Wall-Targeting Antibiotics, but Not Antiseptics

As the RNA-Seq results implicated the role of σ^B^ in cell wall synthesis, we therefore explored the implication of σ^B^ in the susceptibility of mastitis-causing *S. aureus* to cell-wall-targeting antibiotics. The wild-type RF122 and the Δ*sigB* mutant strain were subjected to disk diffusion and microdilution assay to determine their susceptibility against cell-wall-targeting antibiotics as well as commonly used antibiotics and antiseptics for bovine mastitis treatments. The results of the antibiotic susceptibility assay indicated that the Δ*sigB* mutant and the wild type have the same antibiotic profile (Table 2) to cell-wall-targeting β-lactam antibiotics such as ampicillin, penicillin G, cephalothin, cefoxitin, ceftiofur, and oxacillin. The results of the tested antiseptics, iodine, and chlorhexidine, show that both the wild-type and Δ*sigB* mutant strains have comparable MIC values of 0.005% iodine and 0.00002% chlorhexidine. The MBC values of iodine and chlorhexidine were 0.01 and 0.0002% in both strains, respectively.

**Table 2:** Antibiotic susceptibility test of wild type and Δ*sigB* strain.

**Antibiotics**	**Average inhibition zone diameter (mm)**			
	**Wild type**	**Interpretation^#^**	**Δ*sigB***	**Interpretation^#^**
Ampicillin (10 μg)	32	S	35	S
Penicillin G (10 μg)	38.5	S	42	S
Oxacillin (1 μg)	22	S	27	S
Cefoxitin (30 μg)	25	S	30	S
Ceftiofur (30 μg)	26.5	S	30	S
Cephalothin (30 μg)	36	S	41.5	S
Tetracycline (30 μg)	24	S	24	S
Novobiocin (30 μg)	27.5	S	28	S
Trimethoprim–sulfamethoxazole (25 μg)	23.5	S	24	S
Sulfamethoxazole (25 μg)	15	I	15	I
Erythromycin (15 μg)	20.5	I	20.5	I
Streptomycin (10 μg)	10.5	R	10	R

# *The interpretations follow those by the Clinical and Laboratory Standards Institute (CLSI, 2012) except ceftiofur (Therapeutic, 2007) and streptomycin (Shittu and Lin, 2006). S, susceptible; I, intermediate; R, resistant. The experiments were performed in biological duplicates*.

### σ^B^ Plays a Role in Hydrogen Peroxide Stress but Not CHP Stress

The σ^B^ proteins of other Gram-positive bacteria such as *Bacillus subtilis* and *Listeria monocytogenes* are involved in oxidative stress response (Hecker et al., 2007). Here, we also investigated the role of σ^B^ in response to oxidative stressors. The wild-type and Δ*sigB* mutant strains were assessed for their abilities to survive upon exposure to oxidants. The solution of 880 mM H_2_O_2_ was used as an oxidant with exposure times of 10 and 20 min. The results are shown in Table 3. Log reduction values of the Δ*sigB* mutant at 10 and 20 min are statistically different when compared to those of the wild type (*p* < 0.05). This finding correlates with previous studies in that σ^B^ contributes to the ability to tolerate H_2_O_2_ (Kullik et al., 1998; Giachino et al., 2001).

**Table 3:** Summary of phenotypic characterization of wild type and Δ*sigB* mutant.

**Strain**	**Average log reduction ± SD**						
	**880 mM H**_****2****_**O**_****2****_		**7.5 mM CHP**			**Biofilm formation (OD**_****595****_**)**	
	**10 min**	**20 min**	**15 min**	**30 min**	**60 min**	**24 h**	**48 h**
Wild type	2.033 ± 0.419	1.668 ± 0.319	0.114 ± 0.088	0.206 ± 0.140	0.306 ± 0.168	0.123 ± 0.026	0.122 ± 0.024
Δ*sigB*	5.670^*^± 3.018	3.614^*^± 1.352	0.211 ± 0.038	0.362 ± 0.04	0.484 ± 0.095	0.140 ± 0.044	0.097^*^± 0.026

* *Statistical differences are reported if p < 0.05*.

The ability of wild-type and Δ*sigB* mutant strains to survive inorganic oxidant stress, 7.5 mM CHP, was also determined. Log reduction values of both strains are presented in Table 3. Log reduction values between wild-type and Δ*sigB* mutant strains at 15, 30, and 60 min are not statistically significant.

### σ^B^ Is Important to Biofilm Formation but Does Not Influence Cell Invasion and Hemolysis Activity in RF122

The ability of bacteria to form biofilm could suggest the cause of chronic mastitis. Hence, the biofilm formation assay was performed in the wild-type and Δ*sigB* mutant strains. As shown in Table 3, at 24 h, the wild type and Δ*sigB* mutant could form biofilm in comparable extent. However, at 48 h, the Δ*sigB* mutant formed significantly less biofilm than the wild-type strain (*p* = 0.038). Our results support and correlate with the results of previous studies that σ^B^ contributes to biofilm formation (Lauderdale et al., 2009; Mitchell et al., 2010).

We also investigated the ability of Δ*sigB* mutant and wild-type strains to invade the host cells. Both strains did not show significant difference in the ability to invade host cells (data not shown, *p* = 0.11). In addition, we also looked into the ability of the wild-type and Δ*sigB* mutant strains to lyse red blood cells. We found that the hemolytic activities of wild-type and Δ*sigB* mutant strains were also not significantly different; the hemolytic units of the wild type and Δ*sigB* mutant were 4.

## Discussion

σ^B^ in *S. aureus* is involved in stress response and highly expressed during the stationary phase (Hecker et al., 2007). In previous studies, most of the *sigB* mutant strains were constructed by insertion mutations, and inactivation of σ^B^ was done through disruption of a gene encoding regulator of sigma B *rsbU* located upstream of the *rsbV*–*rsbW*–*sigB* operon (Bischoff et al., 2004; Tuchscherr et al., 2015). The growth characteristics of Δ*sigB* mutants in these other backgrounds (e.g., human isolate Newman and murine isolate LS1) were comparable to those of their wild-type strains (Bischoff et al., 2004; Tuchscherr et al., 2015). In contrast, in this study, the Δ*sigB* mutant strain was constructed by an in-frame deletion of the *sigB* gene. Our Δ*sigB* mutant strain showed impaired growth in comparison to the RF122 wild type. The reason comparable growth characteristics were found between the wild type and its Δ*sigB* mutant in previous studies could be due to the leaky activity of σ^B^ from its intact *sigB* gene under the activation pathway independent of RsbU. In addition to change in growth characteristics, RF122 Δ*sigB* also appears off-white. σ^B^ has been shown to positively regulate the *SAB2176* gene, which encodes a secretory antigen staphyloxanthin precursor. Lower expression of this gene in the Δ*sigB* mutant could correlate to less production of the yellowish pigment on its colony (Supplementary Figure 1).

### Effect of σ^B^ on Other Virulence Regulators

The well-known virulence regulators in *S. aureus* are Agr, SarA, SaeRS, ArlRS, and σ^B^ (Jenul and Horswill, 2018). Transcriptome profiles in this study show a positive influence of σ^B^ on SarA, which is similar to other studies (Bischoff et al., 2004). Bischoff et al. (2001) found that σ^B^ has a negative influence on expression of RNAIII, which is an *agr* effector molecule (Bischoff et al., 2001). From our transcriptome analysis, no significant changes in *agr* operon expression in the wild type and Δ*sigB* mutant strains were observed. In RF122, σ^B^ negatively affected expression of *SAB1004* (RNAIII), but the fold change is <2 (Supplementary Table 3). These findings suggest that the *agr* in RF122 is not directly regulated by σ^B^ like other strains (Bischoff et al., 2001; Figueiredo et al., 2017). However, similar to previous findings, our RNA-Seq and qRT-PCR results show that *sarA* are upregulated by σ^B^ and that σ^B^ has a negative effect on several genes encoding exotoxins and secreted enzymes. These genes include staphylococcal exotoxin genes (*set2,3,4,5.7*), which are positively regulated by the *yabJ-spoVG* operon and lipase (*lip*) (Supplementary Table 3) (Meier et al., 2007; Schulthess et al., 2011). In comparison to human clinical isolates (Bischoff et al., 2004), the influence of σ^B^ on virulence genes varies among *S. aureus* strains (Table 4). The differences in σ^B^ regulons might be due to the different nature of bacterial strains. Our invasion assay showed no effects of σ^B^ during invasion, which is similar to the result in the LS1 strain (Tuchscherr et al., 2015). In *S. aureus* LS1, σ^B^ is responsible for intracellular persistence, whereas *sarA* and *agr* are involved in invasion and infection of the strain (Tuchscherr et al., 2015).

**Table 4:** List of virulence genes affected by σ^B^ in this study in comparison to Bischoff et al. (2004).

**Gene**	**Function**	**Regulated by σ**^**B**^	
		**This study**	**Bischoff et al. (2004)**
*capA, capB, capC, capD, capF, capG, cap8H, cap8I, capL, capM, capN, capO, capP*	Capsular polysaccharide synthesis enzyme	+	+ to *cap5A, cap5B, cap5C, cap5D, cap5E, cap5F, cap5G, cap5H, cap5I, cap5J, cap5L, cap5M, cap5O*
*femA*	Factor essential for expression of methicillin resistance	+	Not reported
*femB*	Methicillin resistance factor protein	+	N
*SAB0786*	Hemolysin	+	Not reported
*hla*	α-Hemolysin precursor	–	–
*seg*	Enterotoxin G	+	Not reported
*sen*	Enterotoxin N	+	Not reported
*sec-variant*	Enterotoxin type C variant	+	Not reported
*coa*	Coagulase	N	+
*fib*	Fibrinogen-binding protein	+	Not reported
*clfA*	Clumping factor A	+	+
*SAB0568*	Lipase	+	–

*+, positively regulated by σ^B^; –, negatively regulated by σ^B^; N, not affected by σ^B^; Not reported, not reported as σ^B^-regulated in Bischoff et al. (2004)*.

### Reduction of Biofilm Production Related to Lack of σ^B^

With regard to the ability of the pathogen to form biofilm, our finding with the bovine mastitis RF122 strain correlates with previous findings in MA12 and Newbould strains (Rachid et al., 2000; Lorenz et al., 2008; Mitchell et al., 2010). At 48 h under the experimental conditions (TSB containing 0.5% glucose), the Δ*sigB* mutant showed a significant decrease in biofilm formation when compared to the wild type, indicating that σ^B^ contributes to biofilm formation in RF122. Biofilm is composed of extracellular polymeric substances (EPS) while major compositions are polysaccharides, proteins, nucleic acid, and lipids (Flemming and Wingender, 2010). Several capsular polysaccharide synthesis genes (*cap*) have reduced expression levels in the Δ*sigB* mutant. A strong correlation between the capsule genotype/phenotype and the amount of formed biofilm were identified (Salimena et al., 2016). In *S. aureus*, biofilm formation is related to production of polysaccharide intercellular adhesion (PIA), which depends on the expression of the *icaADBC* operon (Valle et al., 2003). However, based on our RNA-Seq results, σ^B^ did not influence the expression of *icaA*. We propose that the role of σ^B^ on biofilm formation in mastitis-causing *S. aureus* is *ica* independent. Figueiredo et al. (2017) summarized *ica*-independent biofilm formation genes, and some of those previously reported genes were also identified to be σ^B^ dependent in this study, i.e., *clfA, hla*, and *SAB1679c* (serine protease precursor).

### σ^B^ Plays a Minor Role in Resistance to Antiseptics and Antibiotics Used in Dairy Farms

In our study, the *sigB* mutant and its wild type were susceptible to β-lactams. However, the *sigB* mutant showed wider clear zones, in comparison to the wild type, when exposed to the drugs. The phenotypes suggest the role of σ^B^ in resistance to β-lactam antibiotics. In terms of gene expression, *murG* and *murC*, which are involved in biosynthesis of murein sacculus and peptidoglycan, were positively regulated by σ^B^. This is in agreement with previous findings which reported that cell-wall-targeting antibiotics such as β-lactams and vancomycin as well as DNA-synthesis-targeting antibiotics such as sulfamethoxazole/trimethoprim can activate σ^B^ in vancomycin resistance strains (VRSA) (Chen et al., 2011).

Povidone-iodine and chlorhexidine are commonly used antiseptics in dairy cows. We therefore determined the susceptibility of wild-type and Δ*sigB* mutant strains to iodine and chlorhexidine. MICs of povidone-iodine in wild-type and Δ*sigB* mutant strains are equivalent (0.005%). Interestingly, the MIC is still lower than the recommended concentration (0.1%) used in dairy farms (Azizoglu et al., 2013). Similar to the results of povidone-iodine, the MIC of chlorhexidine antiseptic in Δ*sigB* mutant strain is also comparable (0.0002%) to that of the wild type. The MIC was identical to the previously reported value (0.0002%) by Azizoglu et al. (2013) when tested with seven other mastitis *S. aureus* strains. The results suggested that σ^B^ is not involved in the ability of *S. aureus* to tolerate commonly used antiseptics such as povidone-iodine and chlorhexidine.

Previous reports showed that the Δ*sigB* mutant strain of human isolates (ATCC12598, FDA486, SH1000, LAC, Newman, GP268, and BB255) had significant increases in α-hemolysin production in comparison to their wild-type strains (Giachino et al., 2001; Palma and Cheung, 2001; Lauderdale et al., 2009; Chen et al., 2011). σ^B^ negatively regulates *hla* (α-hemolysin) expression (Bischoff et al., 2004; Chen et al., 2011). However, bovine *S. aureus* isolates including RF122 have been reported to produce higher levels of α-toxin than human *S. aureus* isolates perhaps due to higher levels of *agrA, arlR, saeR*, and *sarZ* (positive regulators of *hla*) expression and lower levels of *rot* (negative regulator of *hla*) expression (Liang et al., 2011). To assess the role of σ^B^ in pathogenicity, hemolysis assays and biofilm formation assays were performed. Our study demonstrated that the RF122 Δ*sigB* mutant and wild-type strains could lyse the sheep red blood cells to a comparable extent, suggesting that σ^B^ may play a trivial role in α-hemolytic activity in *S. aureus*-causing mastitis RF122 strain.

### σ^B^ Plays a Role in General Stress Responses

RNA-Seq and qRT-PCR results confirm that σ^B^ directly regulates the expression of alkaline shock protein (*asp23*) in response to general stress. On the contrary, Schulthess et al. (2011) showed that *asp23* is downregulated by *yabJ-spoVG* and upregulated by σ^B^. For oxidative stress response, σ^B^ positively controls *sodA* and *katA* expressions, and the phenotype of the Δ*sigB* mutant was significantly different from that of the wild type upon H_2_O_2_ exposure. In contrast, CHP (organic hydroperoxide) had no effect on the survival of the Δ*sigB* mutant strain compared to the wild-type strain. Perhaps, the regulators or genes which respond to CHP (organic hydroperoxide) stress are not regulated by σ^B^. In *Mycobacterium smegmatis*, a regulator responding to organic hydroperoxide is OhrR, a MarR type of transcriptional regulator (Saikolappan et al., 2015). Although a regulator similar to OhrR was not found in this study, none of the MarR transcriptional regulator family is influenced by σ^B^.

### Newly Identified Genes Regulated by σ^B^

Our transcriptomic result identified newly identified σ^B^-dependent genes. Most of these genes are located in operons, which are the *murQ*-PTS system (SAB0131-0132), *nrdG-nrdD* (SAB2490c-2491c), and *yabJ-spoVG* (SAB0446-0447). Kozytska et al. (2010) identified a list of genes which are found only in *S. aureus* bovine strains. Among the bovine strain genes, two genes (*SAB1880* and *SAB1889*) are shown to be regulated by σ^B^ in our study. The finding suggests that the role of σ^B^ is diverse among *S. aureus* strains.

## Conclusion

This RNA-Seq study demonstrates that the alternative sigma factor B in bovine, the *S. aureus* mastitis RF122 strain, contributes to the expression of several virulence determinants and stress responses. RF122 σ^B^ is important for biofilm formation and H_2_O_2_ response while playing a minor role in α-hemolytic activity and tolerance against iodine, chlorhexidine, and alkyl hydroperoxide stresses. Even though σ^B^ does not contribute to invasion of this bacterial strain, other studies suggested that σ^B^ is important to intracellular survival. The biofilm formation and intracellular persistence are important factors for mastitis onset. For future studies, the roles of σ^B^ in biofilm formation and intracellular persistence are to be elucidated. Better understanding of *S. aureus* persistence mechanisms in bovine mastitis could validate σ^B^ as a potential drug target.

## Data Availability Statement

The datasets generated for this study can be found in the NCBI bioproject accession number PRJNA548307 (https://www.ncbi.nlm.nih.gov/bioproject/PRJNA548307).

## Author Contributions

SS collaborated in the experimental designs, performed parts of the experiments, analyzed the results, and prepared the manuscript. PM performed parts of the experiments, analyzed the result, and prepared the manuscript. PS assisted with mutant construction. PC and WN performed invasion assay. CJ supervised the invasion assay and prepared the manuscript. SC designed and supervised the experiments and the results analyses and prepared the manuscript.

### Conflict of Interest

The authors declare that the research was conducted in the absence of any commercial or financial relationships that could be construed as a potential conflict of interest.
