# Supplementary material for: Alternative Sigma Factor B in Bovine Mastitis-Causing Staphylococcus aureus: Characterization of Its Role in Biofilm Formation, Resistance to Hydrogen Peroxide Stress, Regulon Members
Source: Front Microbiol. 2019 Nov 7;10:2493. doi: 10.3389/fmicb.2019.02493 (PMC6853994; doi:10.3389/fmicb.2019.02493)
Supplement: Supplementary file 1 [file Table_1.DOCX]

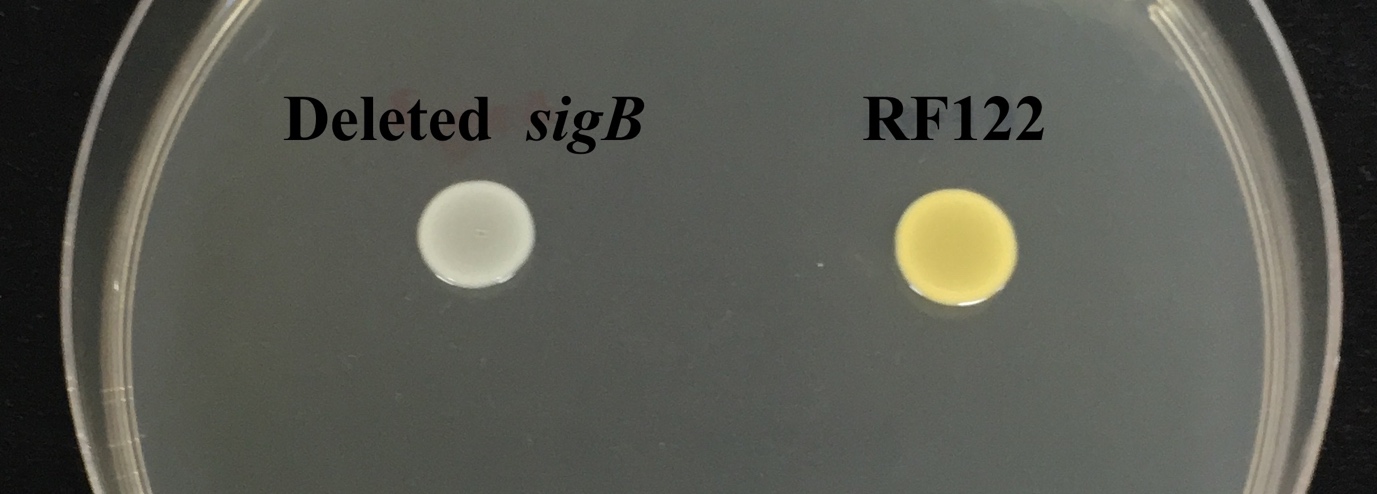


**Supplementary Figure 1** Macroscopic characteristic of *S. aureus* RF122 and Δ*sigB* mutant strains on TSA. The Δ*sigB* mutant shows less yellow pigment (potentially staphyloxanthin production) in comparison to wild type strain.
