# Supplementary material for: Alternative Sigma Factor B in Bovine Mastitis-Causing Staphylococcus aureus: Characterization of Its Role in Biofilm Formation, Resistance to Hydrogen Peroxide Stress, Regulon Members
Source: Front Microbiol. 2019 Nov 7;10:2493. doi: 10.3389/fmicb.2019.02493 (PMC6853994; doi:10.3389/fmicb.2019.02493)
Supplement: Supplementary file 2 [file Table_2.DOCX]

**Supplementary Table 1** Primers and probes used in this study.

| **Primer names** | **DNA sequences (5' -> 3')** |
| --- | --- |
| **Cloning** | |
| SigB-Up1 (*Bam*HI) | TAACATGGATCCTAAAGAAGATTTTATCGAAATGCG |
| SigB-Up2 | ATGTGCTGCTTCTGAAACTTCATTAGCTGATTTCG |
| SigB-Dw1 | AATGAAGTTTCAGAAGCAGCACATAAATAGAATTTGC |
| SigB-Dw2 (*Sal*I) | ATTAATGTCGACATTGTCCATTTACTTAACCCACAC |
| SigB-Ex1 | ACACCTATGAGACAAGATGGAACTC |
| SigB-Ex2 | CCTCAGTAAGTAATCCTTGCTGTTC |
| **qRT-PCR** | |
| rpoB-Fw | GCGCACATGGTTGATGATAAATT |
| rpoB-Rev | CGCCAAGTGGTTGTTGTGTAA |
| asp23-Fw | GTATCGCTGCACGTGAAGTTAAAG |
| asp23-Rev | TTTCACCAACTTCAACAGATACACCT |
| yabJ-Fw | TCGCATGCAACAGTTGTAAATG |
| yabJ-Rev | GATCCTGCTTCTTCCAAAACAATC |
| sarA-Fw | CGTAATGAGCATGATGAAAGAACTGT |
| sarA-Rev | TTGCTTCAGTGATTCGTTTATTTACTC |
| catalase-Fw | AATGTTTGCCCGTTTTTCTACTG |
| catalase-Rev | TGTCACGCTCCGCATCAG |
| sodA-Fw | CACCAAACTCAGAAGAAAAAGGTACTG |
| sodA-Rev | CAAGCCCAACCTGAACCAA |
| SAB2006c-Fw | ACGTCGCGGAGATGATTTAGTT |
| SAB2006c-Rev | GTCGCAAATGCTGCTGCTAAT |
| nrdD-Fw | GGCAACGCGAATGTAACTTCA |
| nrdD-Rev | AGCCACCATATTGACTGCTAGAAA |
| **Probes** | |
| rpoB | FAM-TGCGCGTTCAACAGGACCATATTCAC-BHQ1 |
| asp23 | FAM-CTTAACTGATACATTCACTAATGCATTCTCAAGTGGCAA-BHQ1 |
| yabJ | FAM-AAGCGCTGATGTTCAAGCACAGACAAAAC-BHQ1 |
| sarA | FAM-TGTTAATGCACAACAACGTA-BHQ1 |
| catalase | FAM-AGCAGGAGAACGTGGTG-BHQ1 |
| sodA | FAM-AATTTGCTGACAAAGCAGCTGCACGC-BHQ1 |
| SAB2006c | FAM-CACCGTGGAACTTCCCTACAAACCAAACA-BHQ1 |
| nrdD | FAM-AAAATCAATACAAACTGCATCAGCGCAGC-BHQ1 |
