# Supplementary material for: Alternative Sigma Factor B in Bovine Mastitis-Causing Staphylococcus aureus: Characterization of Its Role in Biofilm Formation, Resistance to Hydrogen Peroxide Stress, Regulon Members
Source: Front Microbiol. 2019 Nov 7;10:2493. doi: 10.3389/fmicb.2019.02493 (PMC6853994; doi:10.3389/fmicb.2019.02493)
Supplement: Supplementary file 3 [file Table_3.DOCX]

| **Sample** | **Total base** | **Total read** | **Mapped read** | **Mean length** |
| --- | --- | --- | --- | --- |
| Wild type (Replicate A) | 61,915,221 | 24,893,366 | 22,927,007 | 101 |
| Wild type (Replicate B) | 53,817,921 | 25,111,395 | 23,004,297 | 101 |
| Wild type (Replicate C) | 56,598,261 | 25,602,763 | 23,419,494 | 101 |
| Δ*sigB* mutant (Replicate A) | 55,805,215 | 24,334,432 | 22,426,079 | 101 |
| Δ*sigB* mutant (Replicate B) | 55,589,289 | 24,685,499 | 22,713,869 | 101 |
| Δ*sigB* mutant (Replicate C) | 82,161,011 | 28,071,246 | 25,768,837 | 101 |

**Supplementary Table 2** Detail of RNAseq result.
