# Supplementary material for: Alternative Sigma Factor B in Bovine Mastitis-Causing Staphylococcus aureus: Characterization of Its Role in Biofilm Formation, Resistance to Hydrogen Peroxide Stress, Regulon Members
Source: Front Microbiol. 2019 Nov 7;10:2493. doi: 10.3389/fmicb.2019.02493 (PMC6853994; doi:10.3389/fmicb.2019.02493)
Supplement: Supplementary file 4 [file Table_4.DOCX]

**Supplementary Table 3** List of genes affected by sigma B as organized by functional classes. *Up-regulated gene is regular number. Down-regulated gene is number with minus sign.

| Gene | Class | Subclass | Gene symbol | Description | Foldchange | *p*adj |
| --- | --- | --- | --- | --- | --- | --- |
| SAB0055 | Amino acid biosynthesis | Serine family |  | Cysteine synthase A protein | -2.203 | 0.000 |
| SAB1938 | Amino acid biosynthesis | Pyruvate family |  | Dihydroxyacid dehydratase | -2.123 | 0.000 |
| SAB0420c | Amino acid biosynthesis | Glutamate family |  | Transcriptional activator of glutamate synthase operon | -2.115 | 0.000 |
| SAB0307c | Amino acid biosynthesis | Aspartate family | *metE2* | 5-methyltetrahydrofolate-homocysteine methyltransferase | -2.108 | 0.000 |
| SAB0308c | Amino acid biosynthesis | Aspartate family |  | Cystathionine beta-lyase | -2.088 | 0.000 |
| SAB0122c | Amino acid biosynthesis | Glutamate family | *argB* | Acetylglutamate kinase | -2.079 | 0.000 |
| SAB0309c | Amino acid biosynthesis | Aspartate family |  | Cystathionine gamma-synthase | -2.061 | 0.000 |
| SAB1939 | Amino acid biosynthesis | Pyruvate family | *ilvB* | Acetolactate synthase large subunit | -2.051 | 0.000 |
| SAB0123c | Amino acid biosynthesis | Glutamate family | *argJ* | Arginine biosynthesis bifunctional protein | -2.032 | 0.000 |
| SAB1880c | Amino acid biosynthesis | Serine family | *hemA* | 5-aminolevulinic acid synthase | 2.083 | 0.000 |
| SAB1171 | Amino acid biosynthesis | Serine family | *glnR* | Glutamine synthetase transcription repressor | 4.585 | 0.000 |
| SAB0462 | Amino acid biosynthesis | Serine family |  | Cysteine synthase | 6.831 | 0.000 |
| SAB2146 | Biosynthesis of cofactors, prosthetic groups, and carriers | Molybdopterin | *moaC* | Molybdenum cofactor biosynthesis protein | -2.335 | 0.000 |
| SAB0774 | Biosynthesis of cofactors, prosthetic groups, and carriers | Other |  | ATP-binding ABC transporter protein | 2.054 | 0.000 |
| SAB1281c | Biosynthesis of cofactors, prosthetic groups, and carriers | Folic acid | *dfrB* | Trimethoprim-sensitive dihydrofolate reductase | 2.850 | 0.000 |
| SAB0278c | Cell envelope | Other |  | Membrane protein | -2.451 | 0.000 |
| SAB0290c | Cell envelope | Other |  | Membrane protein | -2.056 | 0.000 |
| SAB0054c | Cell envelope | Iron related metabolism | *sirA* | Iron-regulated lipoprotein | -2.046 | 0.000 |
| SAB1649 | Cell envelope | Other |  | Conserved hypothetical protein | -2.040 | 0.000 |
| SAB1633c | Cell envelope | Biosynthesis and degradation of surface polysaccharides and lipopolysaccharides |  | Exported protein | 2.004 | 0.018 |
| SAB0895 | Cell envelope | Other |  | Exported protein (pseudogene) | 2.017 | 0.000 |
| SAB0097 | Cell envelope | Biosynthesis and degradation of surface polysaccharides and lipopolysaccharides | *cap8H* | Capsular polysaccharide synthesis enzyme CapH | 2.021 | 0.000 |
| SAB0101 | Cell envelope | Biosynthesis and degradation of surface polysaccharides and lipopolysaccharides | *capL* | Capsular polysaccharide synthesis enzyme CapL | 2.037 | 0.000 |
| SAB1215 | Cell envelope | Other |  | Membrane protein | 2.051 | 0.000 |
| SAB0894 | Cell envelope | Other |  | Membrane protein | 2.055 | 0.000 |
| SAB0566 | Cell envelope | Other |  | Exported protein | 2.064 | 0.000 |
| SAB0093 | Cell envelope | Biosynthesis and degradation of surface polysaccharides and lipopolysaccharides | *capD* | Capsular polysaccharide synthesis enzyme CapD | 2.083 | 0.000 |
| SAB1020 | Cell envelope | Other |  | Membrane protein | 2.088 | 0.000 |
| SAB2019c | Cell envelope | Other |  | Membrane protein | 2.090 | 0.000 |
| SAB0653 | Cell envelope | Biosynthesis and degradation of surface polysaccharides and lipopolysaccharides |  | Bactoprenol glucosyl transferase or stress response regulator | 2.122 | 0.000 |
| SAB1691 | Cell envelope | Other |  | Exported protein | 2.134 | 0.000 |
| SAB1273c | Cell envelope | Biosynthesis of murein sacculus and peptidoglycan | *murG* | UDP-N-acetylglucosamin--N-acetylmuramyl-(pentapeptide) pyrophosphoryl-undecaprenol N-acetylglucosamine transferase | 2.176 | 0.000 |
| SAB0567 | Cell envelope | Other |  | Membrane protein | 2.179 | 0.000 |
| SAB0615 | Cell envelope | Other |  | Membrane protein | 2.184 | 0.000 |
| SAB0896 | Cell envelope | Other |  | Membrane protein | 2.208 | 0.000 |
| SAB1600c | Cell envelope | Biosynthesis of murein sacculus and peptidoglycan | *murC* | UDP-N-acetylmuramate-L-alanine ligase | 2.275 | 0.001 |
| SAB0897 | Cell envelope | Other |  | Exported protein | 2.330 | 0.000 |
| SAB1343c | Cell envelope | Biosynthesis and degradation of surface polysaccharides and lipopolysaccharides | *ebpS* | Cell surface elastin binding protein | 2.425 | 0.000 |
| SAB0104 | Cell envelope | Biosynthesis and degradation of surface polysaccharides and lipopolysaccharides | *capO* | Capsular polysaccharide synthesis enzyme CapO | 2.605 | 0.000 |
| SAB1261 | Cell envelope | Other |  | Membrane protein | 2.613 | 0.025 |
| SAB0742 | Cell envelope | Other |  | Membrane protein | 2.632 | 0.000 |
| SAB1788c | Cell envelope | Other |  | Membrane protein | 2.689 | 0.000 |
| SAB1664c | Cell envelope | Other |  | Exported protein | 2.694 | 0.000 |
| SAB0105 | Cell envelope | Biosynthesis and degradation of surface polysaccharides and lipopolysaccharides | *capP* | Capsular polysaccharide synthesis enzyme CapP | 2.766 | 0.000 |
| SAB0901 | Cell envelope | Biosynthesis and degradation of surface polysaccharides and lipopolysaccharides |  | Glycosyl transferase | 2.813 | 0.000 |
| SAB0919c | Cell envelope | Biosynthesis and degradation of surface polysaccharides and lipopolysaccharides | *atl* | Autolysin | 2.915 | 0.000 |
| SAB0096 | Cell envelope | Biosynthesis and degradation of surface polysaccharides and lipopolysaccharides | *capG* | Capsular polysaccharide synthesis enzyme CapG | 2.924 | 0.000 |
| SAB0095 | Cell envelope | Biosynthesis and degradation of surface polysaccharides and lipopolysaccharides | *capF* | Capsular polysaccharide, synthesis enzyme CapF | 3.028 | 0.000 |
| SAB0969 | Cell envelope | Other |  | Membrane protein | 3.262 | 0.000 |
| SAB0098 | Cell envelope | Biosynthesis and degradation of surface polysaccharides and lipopolysaccharides | *cap8I* | Capsular polysaccharide synthesis enzyme CapI | 3.555 | 0.000 |
| SAB1078c | Cell envelope | Other |  | Membrane protein | 4.951 | 0.000 |
| SAB0726 | Cell envelope | Other |  | Exported protein | 6.183 | 0.000 |
| SAB0102 | Cell envelope | Biosynthesis and degradation of surface polysaccharides and lipopolysaccharides | *capM* | Capsular polysaccharide synthesis enzyme CapM | 6.192 | 0.000 |
| SAB0091 | Cell envelope | Biosynthesis and degradation of surface polysaccharides and lipopolysaccharides | *capB* | Capsular polysaccharide synthesis enzyme CapB | 7.796 | 0.000 |
| SAB0103 | Cell envelope | Biosynthesis and degradation of surface polysaccharides and lipopolysaccharides | *capN* | Capsular polysaccharide synthesis enzyme CapN | 9.565 | 0.000 |
| SAB0090 | Cell envelope | Biosynthesis and degradation of surface polysaccharides and lipopolysaccharides | *capA* | Capsular polysaccharide synthesis enzyme CapA | 9.757 | 0.000 |
| SAB2248c | Cell envelope | Other |  | Putative lipoprotein | 10.535 | 0.000 |
| SAB0092 | Cell envelope | Biosynthesis and degradation of surface polysaccharides and lipopolysaccharides | *capC* | Capsular polysaccharide synthesis enzyme CapC | 15.659 | 0.000 |
| SAB2064c | Cell envelope | Other |  | Membrane protein | 41.202 | 0.000 |
| SAB1027c | Cellular | Toxin production | *hla* | Alpha-hemolysin precursor | -3.232 | 0.003 |
| SAB1229 | Cellular | Resistance mechanisms | *femA* | Factor essential for expression of methicillin resistance | 2.137 | 0.000 |
| SAB1230 | Cellular | Resistance mechanisms | *femB* | Methicillin resistance factor protein | 2.176 | 0.000 |
| SAB1373c | Cellular | Toxin production |  | Streptolysin-associated protein SagD homolog | 2.197 | 0.000 |
| SAB1172 | Cellular | Resistance mechanisms | *glnA* | Glutamine synthetase | 2.197 | 0.000 |
| SAB1700c | Cellular | Toxin production | *sem-truncated* | Enterotoxin M (pseudogene) | 2.263 | 0.000 |
| SAB1262 | Cellular | Resistance mechanisms |  | Toxic ion resistance protein | 2.298 | 0.005 |
| SAB1697c | Cellular | Toxin production | *sen* | Enterotoxin N | 2.503 | 0.000 |
| SAB2176 | Cellular | Pathogenesis |  | Secretory antigen staphyloxanthin precursor | 2.517 | 0.000 |
| SAB1699c | Cellular | Toxin production | *sei* | Enterotoxin I | 2.549 | 0.000 |
| SAB0786 | Cellular | Toxin production |  | Hemolysin | 2.555 | 0.000 |
| SAB1698c | Cellular | Toxin production | *sec-variant* | Enterotoxin type C variant | 2.661 | 0.000 |
| SAB0675c | Cellular | Resistance mechanisms |  | Nultidrug resistance protein | 2.749 | 0.000 |
| SAB1376c | Cellular | Toxin production |  | Streptolysin S-associated protein SagB homolog | 2.878 | 0.000 |
| SAB1696c | Cellular | Toxin production | *seg* | Enterotoxin G | 2.992 | 0.000 |
| SAB1462c | Cellular process | DNA transformation |  | Competence-related membrane protein | 2.034 | 0.000 |
| SAB1192 | Cellular process | Detoxification |  | Catalase | 4.438 | 0.000 |
| SAB1425c | Cellular process | Detoxification | *sodA* | Superoxide dismutase | 13.799 | 0.000 |
| SAB2529c | Cellular processes | Cell adhesion |  | Surface expressed Ser-Thr rich repeat protein (pseudogene) | -2.362 | 0.000 |
| SAB1290c | Cellular processes | Cell adhesion |  | Truncated cell surface fibronectin-binding protein | -2.069 | 0.000 |
| SAB1021 | Cellular processes | Cell adhesion | *fib* | Fibrinogen-binding protein | 2.049 | 0.000 |
| SAB1022 | Cellular processes | Cell adhesion |  | Fibrinogen-binding protein precursor | 2.156 | 0.000 |
| SAB0872 | Cellular processes | Adaptations to atypical conditions |  | GTP pyrophosphokinase | 3.584 | 0.000 |
| SAB1599c | Cellular processes | Adaptations to atypical conditions |  | General stress response protein | 6.337 | 0.000 |
| SAB2063c | Cellular processes | Adaptations to atypical conditions | *asp23* | Alkaline shock protein | 13.318 | 0.000 |
| SAB0744 | Cellular processes | Cell adhesion | *clfA* | Truncated clumping factor | 17.124 | 0.000 |
| SAB1949c | Cellular processes | Adaptations to atypical conditions | *sigB* | RNA polymerase sigma factor B | 88.239 | 0.000 |
| SAB0252c | Central intermediary metabolism | Amino sugars | *nanA* | N-acetylneuraminate lyase subunit | -2.131 | 0.000 |
| SAB2432c | Central intermediary metabolism | Other |  | D-specific D-2-hydroxyacid dehydrogenase | -2.072 | 0.000 |
| SAB0500c | Central intermediary metabolism | Amino sugars | *amaA* | N-acyl-L-amino acid amidohydrolase | -2.033 | 0.000 |
| SAB2034c | Central intermediary metabolism | Amino sugars | *glmS* | Glucosamine-fructose-6-phosphate aminotransferase | 3.394 | 0.000 |
| SAB1313 | DNA metabolism | DNA replication, recombination, and repair | *recU* | Recombination protein U | 2.273 | 0.018 |
| SAB0272c | Energy metabolism | Glycolysis/gluconeogenesis |  | Trimethylamine dehydrogenase | -2.406 | 0.000 |
| SAB0125c | Energy metabolism | Amino acids and amines | *argD* | Acetylornithine aminotransferase | -2.218 | 0.000 |
| SAB0174c | Energy metabolism | Fermentation |  | Acetyl-CoA/acetoacetyl-CoA transferase | -2.155 | 0.000 |
| SAB0108 | Energy metabolism | Fermentation | *aldA* | Aldehyde dehdydrogenase | -2.041 | 0.000 |
| SAB2517 | Energy metabolism | Sugars | *pmi* | Mannose-6-phosphate isomerase | -2.035 | 0.000 |
| SAB0807 | Energy metabolism | Electron transport |  | NADH dehydrogenase | 2.151 | 0.000 |
| SAB1207 | Energy metabolism | TCA cycle | *citB* | Aconitate hydratase | 2.296 | 0.000 |
| SAB2006c | Energy metabolism | Fermentation |  | Aldehyde dehydrogenase | 2.347 | 0.000 |
| SAB0520 | Energy metabolism | Sugars |  | 3-hexulose-6-phosphate synthase | 3.175 | 0.000 |
| SAB0764 | Energy metabolism | Amino acids and amines | *gcvH* | Glycine cleavage system H protein | 3.184 | 0.000 |
| SAB1109 | Energy metabolism | TCA cycle | *sucC* | Succinyl-CoA synthetase beta chain | 3.247 | 0.000 |
| SAB2186c | Energy metabolism | Anaerobic |  | Formate dehydrogenase alpha subunit | 3.727 | 0.000 |
| SAB2211c | Energy metabolism | Amino acids and amines | *hutG* | Formiminoglutamase | 3.856 | 0.000 |
| SAB2065c | Energy metabolism | Other |  | Membrane-bound oxidoreductase | 55.148 | 0.000 |
| SAB0173c | Fatty acid and phospholipid metabolism | Degradation |  | Acyl-CoA synthetase | -2.363 | 0.000 |
| SAB0172c | Fatty acid and phospholipid metabolism | Degradation | *fadD* | Glutaryl or acyl-CoA dehydrogenase (pseudogene) | -2.304 | 0.000 |
| SAB0171c | Fatty acid and phospholipid metabolism | Degradation |  | 3-hydroxyacyl-CoA dehydrogenase | -2.235 | 0.000 |
| SAB0170c | Fatty acid and phospholipid metabolism | Biosynthesis |  | 3-ketoacyl-CoA transferase | -2.123 | 0.000 |
| SAB0877 | Fatty acid and phospholipid metabolism | Biosynthesis | *fabI* | Trans-2-enoyl-ACP reductase | 2.011 | 0.000 |
| SAB1399c | Fatty acid and phospholipid metabolism | Biosynthesis | *accC* | Acetyl-CoA biotin carboxylase | 2.065 | 0.002 |
| SAB1180 | Fatty acid and phospholipid metabolism | Biosynthesis |  | Cardiolipin synthase | 2.569 | 0.000 |
| SAB0007c | Hypothetical proteins | Conserved |  | SAR0007 | -2.308 | 0.000 |
| SAB2473 | Hypothetical proteins | Conserved |  | SAR2678 | -2.292 | 0.000 |
| SAB2446 | Hypothetical proteins |  |  | SAR2653 | -2.249 | 0.000 |
| SAB0106c | Hypothetical proteins | Hypothetical pseudogenes |  | SAR0167 | -2.157 | 0.000 |
| SAB2452 | Hypothetical proteins |  |  | SAV2578 | -2.144 | 0.000 |
| SAB2447 | Hypothetical proteins | Conserved |  | SAR2654 | -2.116 | 0.000 |
| SAB0443 | Hypothetical proteins | Conserved |  | SAR0495 | -2.090 | 0.007 |
| SAB0826c | Hypothetical proteins | Unique genes |  | Unknown | -2.061 | 0.000 |
| SAB0298c | Hypothetical proteins | Conserved |  | SAV0348 | -2.024 | 0.000 |
| SAB0107c | Hypothetical proteins | Conserved |  | SAR0168 | -2.015 | 0.000 |
| SAB1692 | Hypothetical proteins |  |  | SAR2705 | 2.001 | 0.000 |
| SAB1464c | Hypothetical proteins | Conserved |  | SAR1669 | 2.013 | 0.000 |
| SAB1759 | Hypothetical proteins | Unique genes |  | Unknown | 2.013 | 0.047 |
| SAB0395 | Hypothetical proteins |  |  | SACOL0483 | 2.019 | 0.000 |
| SAB1028 | Hypothetical proteins |  |  | MW1045 | 2.023 | 0.000 |
| SAB1179 | Hypothetical proteins |  |  | SAR1327 | 2.026 | 0.000 |
| SAB1695 | Hypothetical proteins | Unique genes |  | Unknown | 2.037 | 0.000 |
| SAB1888c | Hypothetical proteins | Unique genes |  | Unknown | 2.061 | 0.000 |
| SAB1459c | Hypothetical proteins | Conserved |  | SAR1664 | 2.066 | 0.000 |
| SAB0392 | Hypothetical proteins |  |  | SAV0438 | 2.071 | 0.000 |
| SAB1685c | Hypothetical proteins |  |  | MW1766 | 2.075 | 0.000 |
| SAB1310c | Hypothetical proteins | Conserved |  | SAR1457 | 2.103 | 0.000 |
| SAB1660c | Hypothetical proteins | Conserved |  | SAR1886 | 2.107 | 0.000 |
| SAB0337c | Hypothetical proteins | Conserved |  | SAS0363 | 2.116 | 0.000 |
| SAB0393 | Hypothetical proteins |  |  | SAR0442 | 2.123 | 0.000 |
| SAB0620 | Hypothetical proteins | Conserved |  | SA0626 | 2.133 | 0.000 |
| SAB1674c | Hypothetical proteins |  |  | SAS1737 | 2.138 | 0.000 |
| SAB1689c | Hypothetical proteins | Unique genes |  | Unknown | 2.148 | 0.000 |
| SAB1634c | Hypothetical proteins |  |  | SAR1858 | 2.156 | 0.000 |
| SAB1666c | Hypothetical proteins | Hypothetical pseudogenes |  | MW1749 | 2.164 | 0.000 |
| SAB1878c | Hypothetical proteins | Hypothetical pseudogenes |  | Exiguobacterium sp.drug/metabolite permease superfamily | 2.166 | 0.000 |
| SAB1681c | Hypothetical proteins |  |  | MW1763 | 2.171 | 0.000 |
| SAB1879c | Hypothetical proteins |  |  | Exiguobacterium sp. 255-15 drug permease superfamily | 2.190 | 0.000 |
| SAB1682c | Hypothetical proteins |  |  | SACOL1877 | 2.206 | 0.000 |
| SAB1129 | Hypothetical proteins | Conserved |  | SAR1241 | 2.226 | 0.060 |
| SAB1787c | Hypothetical proteins |  |  | SAR1944 | 2.249 | 0.000 |
| SAB1887c | Hypothetical proteins | Hypothetical pseudogenes |  | SAV0804 | 2.263 | 0.000 |
| SAB1177 | Hypothetical proteins | Conserved |  | SAR1321 | 2.309 | 0.000 |
| SAB0394 | Hypothetical proteins |  |  | SACOL0482 | 2.325 | 0.000 |
| SAB1176 | Hypothetical proteins | Conserved |  | S. epidermidis RP62A SERP0879 | 2.336 | 0.000 |
| SAB1885c | Hypothetical proteins |  |  | SAR2115 | 2.368 | 0.000 |
| SAB0868c | Hypothetical proteins | Conserved |  | SAS0871 | 2.380 | 0.001 |
| SAB0391 | Hypothetical proteins |  |  | SAR0438 | 2.395 | 0.000 |
| SAB1374c | Hypothetical proteins |  |  | *L. monocytogenes* str. 4b F2365 gene LMOf2365_1117 | 2.405 | 0.000 |
| SAB1680c | Hypothetical proteins |  |  | MW1762 | 2.437 | 0.000 |
| SAB1884c | Hypothetical proteins | Mobile element-associated hypothetical proteins |  | SAR2113 | 2.474 | 0.000 |
| SAB0846 | Hypothetical proteins | Conserved |  | SAR0944 | 2.491 | 0.034 |
| SAB1280c | Hypothetical proteins | Conserved |  | SAS1368 | 2.500 | 0.000 |
| SAB1865 | Hypothetical proteins | Conserved |  | SAR2021 | 2.631 | 0.001 |
| SAB1375c | Hypothetical proteins |  |  | *L. monocytogenes* str. 4b F2365 gene LMOf2365_1117 | 2.839 | 0.000 |
| SAB1174 | Hypothetical proteins |  |  | SAR1306 | 2.893 | 0.000 |
| SAB0900 | Hypothetical proteins |  |  | SAR1007 | 2.922 | 0.000 |
| SAB1377c | Hypothetical proteins |  |  | *L. monocytogenes* str. 4b F2365 gene LMOf2365_1116 | 2.952 | 0.000 |
| SAB1173 | Hypothetical proteins |  |  | SAV1312 | 2.975 | 0.000 |
| SAB1676c | Hypothetical proteins |  |  | MW1758 | 3.003 | 0.000 |
| SAB0631c | Hypothetical proteins |  |  | SA0637 | 3.057 | 0.000 |
| SAB1378c | Hypothetical proteins | Unique genes |  | Unknown | 3.211 | 0.000 |
| SAB2363c | Hypothetical proteins |  |  | SAR2569 | 3.230 | 0.001 |
| SAB1665c | Hypothetical proteins | Hypothetical pseudogenes |  | MW1749 | 3.327 | 0.000 |
| SAB0772 | Hypothetical proteins |  |  | SAR0874 | 3.965 | 0.000 |
| SAB1969c | Hypothetical proteins |  |  | SAR2173 | 4.619 | 0.000 |
| SAB0948 | Hypothetical proteins |  |  | MW0964 | 4.827 | 0.000 |
| SAB0743 | Hypothetical proteins | Conserved |  | MW0763 | 4.941 | 0.000 |
| SAB0787 | Hypothetical proteins | Hypothetical pseudogenes |  | SAR0883 | 5.193 | 0.000 |
| SAB1598c | Hypothetical proteins |  |  | SAS1664 | 5.780 | 0.000 |
| SAB1612c | Hypothetical proteins |  |  | SAR1837 | 9.242 | 0.000 |
| SAB2261 | Hypothetical proteins | Conserved |  | SACOL2379 | 9.334 | 0.000 |
| SAB0755c | Hypothetical proteins |  |  | SAR0855 | 17.300 | 0.000 |
| SAB0738 | Mobile and extrachromosomal element functions | Transposon functions |  | Transposase | 2.074 | 0.000 |
| SAB1679c | Mobile and extrachromosomal element functions | Degradation of proteins, peptides, and glycopeptides |  | Serine protease precursor | 2.228 | 0.000 |
| SAB1279c | Mobile and extrachromosomal element functions | Protein modification and repair | *msrA* | Peptide methionine sulfoxide reductase | 2.296 | 0.020 |
| SAB2179 | Mobile and extrachromosomal element functions | Transposon functions |  | Transposase | 2.329 | 0.008 |
| SAB1278c | Mobile and extrachromosomal element functions | Protein modification and repair |  | Peptide methionine sulfoxide reductase | 2.957 | 0.002 |
| SAB1193 | Mobile and extrachromosomal element functions | Ribosomal proteins: synthesis and modification | *rpmG* | 50S ribosomal protein L33 | 3.718 | 0.000 |
| SAB0572 | Mobile and extrachromosomal element functions | Transposon functions |  | Phage recombinase/integrase | 4.127 | 0.000 |
| SAB0078 | Purines, pyrimidines, nucleosides, and nucleotides | Other | *deoB* | Phosphopentomutase | 2.374 | 0.000 |
| SAB1282c | Purines, pyrimidines, nucleosides, and nucleotides | 2'-Deoxyribonucleotide metabolism | *thyA* | Thymidylate synthase | 2.522 | 0.000 |
| SAB2491c | Purines, pyrimidines, nucleosides, and nucleotides | 2'-Deoxyribonucleotide metabolism | *nrdD* | Anaerobic ribonucleotide reductase large subunit | 4.853 | 0.000 |
| SAB0203c | Regulatory functions | DNA interactions |  | Transcriptional regulator GntR family | -2.133 | 0.000 |
| SAB0616 | Regulatory functions | DNA interactions |  | Transcriptional regulator | 2.118 | 0.000 |
| SAB2081c | Regulatory functions | DNA interactions |  | MerR family transcriptional regulator | 2.243 | 0.012 |
| SAB0569c | Regulatory functions | Other | *sarA* | Staphylococcal accessory regulator A | 2.818 | 0.000 |
| SAB0211c | Regulatory functions | DNA interactions |  | Ribose transcriptional repressor LacI family | 3.094 | 0.000 |
| SAB1950c | Transcription | Transcription factors | *rsbW* | Anti-sigma B factor | 2.602 | 0.000 |
| SAB0446 | Transcription | Degradation of RNA | *yabJ* | Translation initiation inhibitor | 2.928 | 0.000 |
| SAB2430 | Transport and binding proteins | Iron and cation-carrying compounds |  | Exported protein | -2.695 | 0.000 |
| SAB0280c | Transport and binding proteins | Carbohydrates, organic alcohols, and acids |  | PTS system component | -2.390 | 0.000 |
| SAB2332 | Transport and binding proteins | Amino acids, peptides and amines |  | Amino acid transporter permease | -2.377 | 0.000 |
| SAB0279c | Transport and binding proteins | Carbohydrates, organic alcohols, and acids |  | PTS system component | -2.363 | 0.000 |
| SAB0281c | Transport and binding proteins | Carbohydrates, organic alcohols, and acids |  | PTS system regulator | -2.298 | 0.000 |
| SAB0251c | Transport and binding proteins | Iron and cation-carrying compounds |  | Transport protein | -2.287 | 0.000 |
| SAB0181c | Transport and binding proteins | Carbohydrates, organic alcohols, and acids |  | Phosphotransferase system enzyme II | -2.270 | 0.000 |
| SAB2431 | Transport and binding proteins | Iron and cation-carrying compounds | *copA* | Copper-transporting ATPase | -2.264 | 0.000 |
| SAB2356 | Transport and binding proteins | Unknown substrate |  | Transport protein | -2.250 | 0.000 |
| SAB0213c | Transport and binding proteins | Membrane transport |  | Membrane transport protein | -2.129 | 0.000 |
| SAB0333c | Transport and binding proteins | Carbohydrates, organic alcohols, and acids |  | Proton/sodium-glutamate symport protein | -2.090 | 0.000 |
| SAB0129c | Transport and binding proteins | Carbohydrates, organic alcohols, and acids | *glcA* | Phosphotransferase system enzyme II glucose-specific factor IIA | -2.056 | 0.000 |
| SAB0059 | Transport and binding proteins | Iron and cation-carrying compounds |  | Siderophore biosynthesis protein, lucA family | -2.043 | 0.000 |
| SAB0053c | Transport and binding proteins | Iron and cation-carrying compounds | *sirB* | Siderophore transport protein | -2.034 | 0.000 |
| SAB0671 | Transport and binding proteins | Amino acids, peptides and amines |  | Glycine betaine/carnitine/choline ATP-binding ABC transport protein | 2.051 | 0.000 |
| SAB2066c | Transport and binding proteins | Amino acids, peptides and amines | *opuD* | Glycine betaine transporter | 2.257 | 0.000 |
| SAB1380c | Transport and binding proteins | Unknown substrate |  | ABC transport protein | 2.423 | 0.000 |
| SAB2245c | Transport and binding proteins | Carbohydrates, organic alcohols, and acids | *lldP* | L-lactate permease | 2.543 | 0.000 |
| SAB0898 | Transport and binding proteins | Unknown substrate |  | ATP-binding ABC transport protein | 2.637 | 0.000 |
| SAB1379c | Transport and binding proteins | Unknown substrate |  | ABC transporter permease | 2.810 | 0.000 |
| SAB1277c | Transport and binding proteins | Carbohydrates, organic alcohols, and acids |  | Phosphotransferase system glucose-specific IIA component | 3.195 | 0.026 |
| SAB1678c | Transport and binding proteins | Unknown substrate |  | Transport protein | 3.934 | 0.000 |
| SAB1677c | Transport and binding proteins | Unknown substrate |  | Transport protein | 4.393 | 0.000 |
| SAB0950 | Transport and binding proteins | Carbohydrates, organic alcohols, and acids | *ptsI* | Phosphoenolpyruvate-protein phosphatase | 5.721 | 0.000 |
| SAB0949 | Transport and binding proteins | Carbohydrates, organic alcohols, and acids | *ptsH* | Histidine-containing phosphocarrier protein | 8.596 | 0.000 |
| SAB0565 | Unknown function | Enzymes of unknown specificity |  | Hydrolase | 2.091 | 0.000 |
| SAB0568 | Unknown function | Enzymes of unknown specificity |  | Esterase or lipase | 2.221 | 0.000 |
| SAB1643c | Unknown function | Enzymes of unknown specificity |  | Aldo-keto reductase family protein | 2.627 | 0.000 |
