# Supplementary material for: Alternative Sigma Factor B in Bovine Mastitis-Causing Staphylococcus aureus: Characterization of Its Role in Biofilm Formation, Resistance to Hydrogen Peroxide Stress, Regulon Members
Source: Front Microbiol. 2019 Nov 7;10:2493. doi: 10.3389/fmicb.2019.02493 (PMC6853994; doi:10.3389/fmicb.2019.02493)
Supplement: Supplementary file 5 [file Table_5.DOCX]

**Supplementary Table 4** Comparison gene expression fold change between wild type and ∆*sigB* strain from RNAseq and qRT-PCR

| Gene | RNAseq  Fold change | qRT-PCR  Fold change |
| --- | --- | --- |
| *asp23* | 13.32 | 7.14 |
| *sarA* | 2.81 | 1.78 |
| *katA* | 4.44 | 1.53 |
| *yabJ* | 2.93 | 3.60 |
| *SAB2006c* | 2.35 | 2.20 |
| *sodA* | 13.80 | 1.60 |
| *nrdD* | 4.85 | 2.88 |

RNAseq, RNA sequencing; qRT-PCR, qualitative real time polymerase chain reaction
